# Supplementary material for: Decadal biomass increment in early secondary succession woody ecosystems is increased by CO2 enrichment
Source: Nat Commun. 2019 Feb 14;10:454. doi: 10.1038/s41467-019-08348-1 (PMC6376023; doi:10.1038/s41467-019-08348-1)
Supplement: Supplementary file 1 — Supplementary Information [file 41467_2019_8348_MOESM1_ESM.pdf]

# Supplementary Information for: Decadal biomass increment in early secondary successional woody ecosystems is increased by $CO_2$ enrichment

Walker et al.

November 10, 2018

## Supplementary Figures

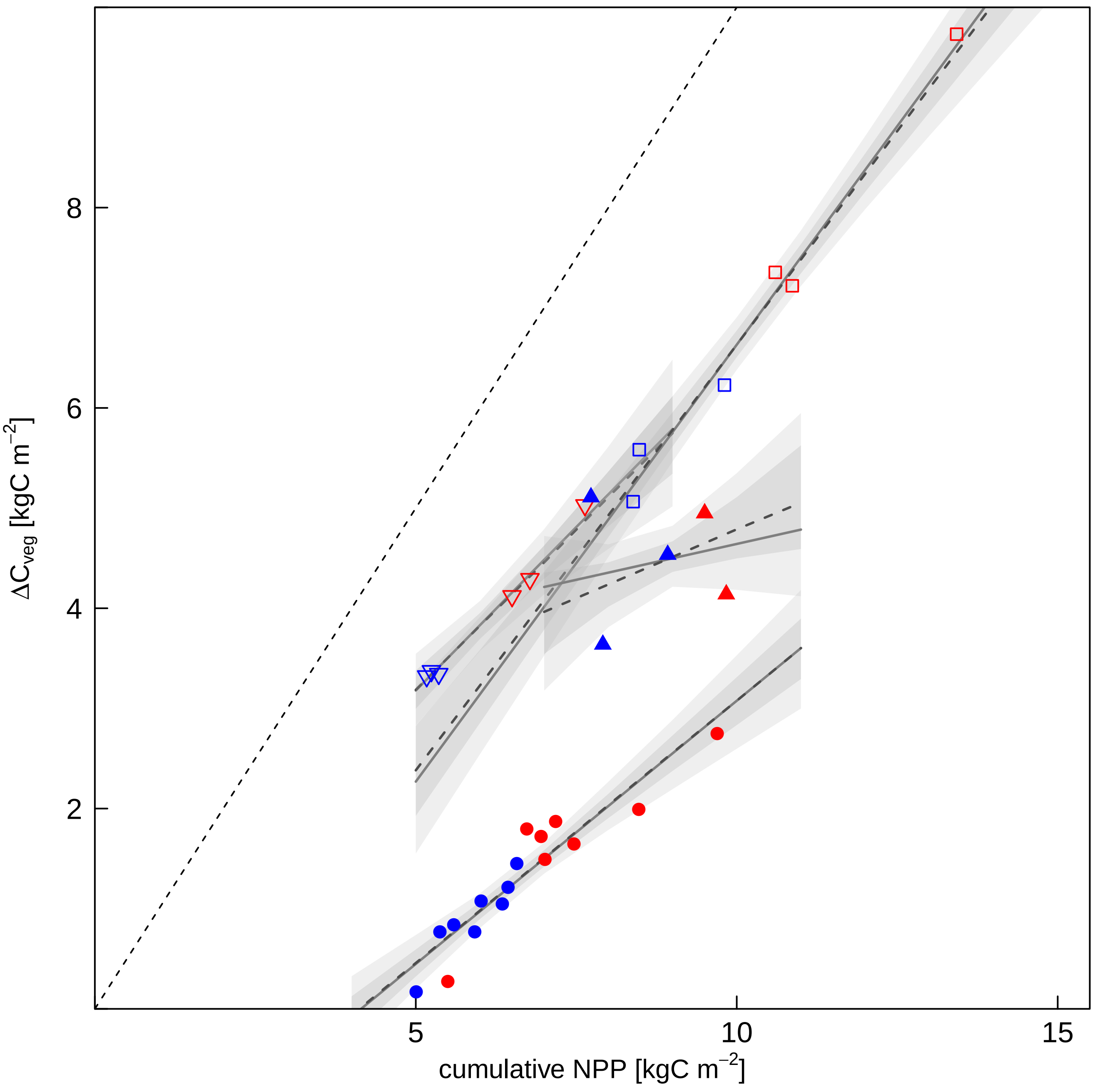


Supplementary Figure 1: Same as Figure 1 but with extended CIs demonstrating strong overlap between Rhinelander and Duke relationships. Also shown are the median values of the boot-strapping which show agreement between the mixed-model fit to the data and the boot-strap median value at Rhinelander, Duke, and KSC, but not at ORNL. This indicates the need for more replicates to be confident in the relationship and $\Delta C_{veg}$ response at ORNL.

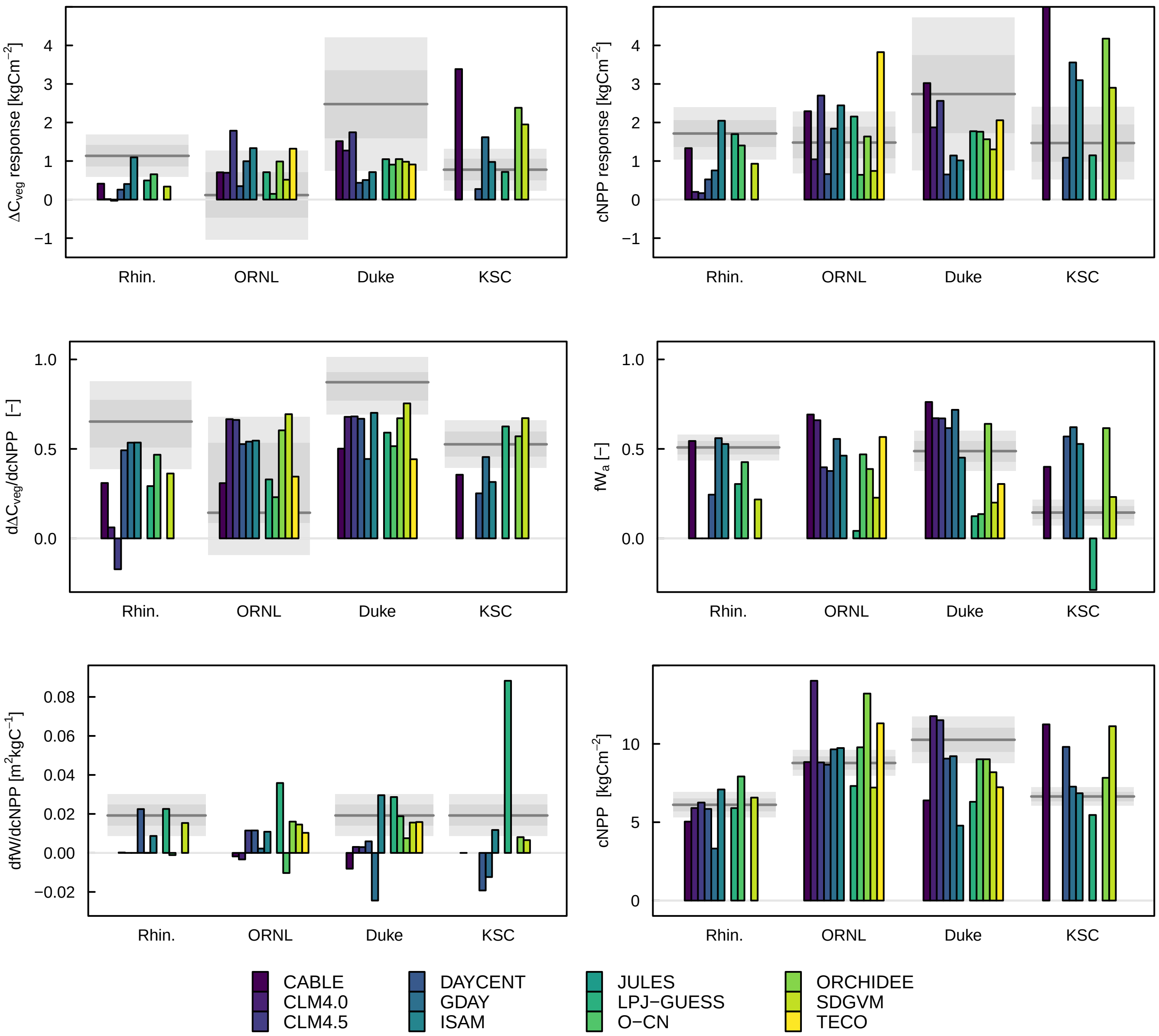


Supplementary Figure 2: As Figure 3 in the main document but individual model predictions of the $\Delta C_{veg}$ response to $CO_2$ enrichment (upper left); the two variables leading to the response—the cNPP response to $CO_2$ enrichment (upper right) and $\frac{d\Delta C_{veg}}{dcNPP}$ (middle left); and the three components of $\frac{d\Delta C_{veg}}{dcNPP}$—$fW_a$ (middle right), $\frac{dfW}{dcNPP}$ (bottom left), and cross-treatment mean cNPP (bottom right). Grey shaded areas represent the observations (dark grey lines represent observed means or regression parameters, light grey polygons represent the ±1 s.e.m. CI and the lighter grey polygons represent the 95 % CI).

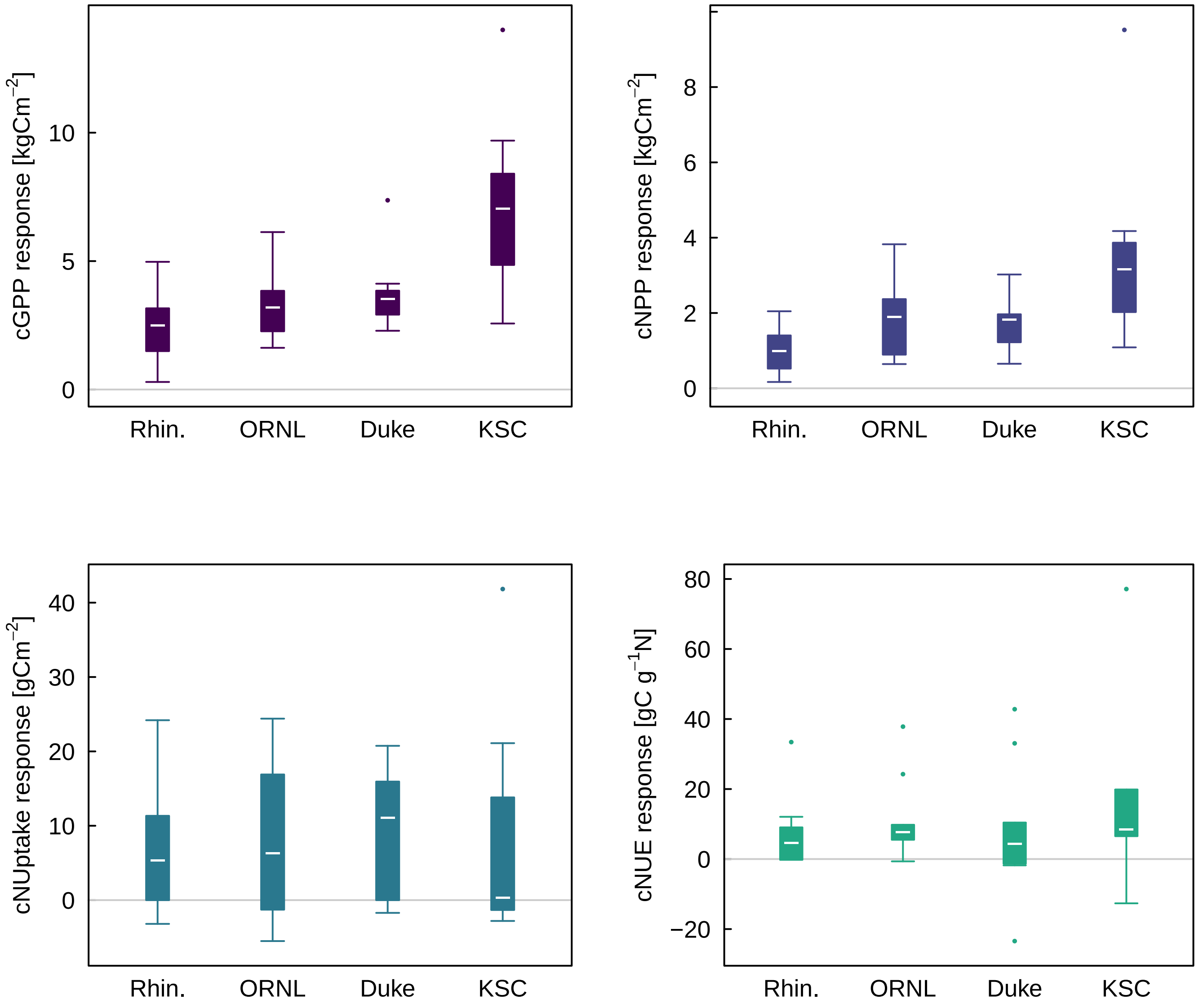


Supplementary Figure 3: Model ensemble predictions of the absolute response to $CO_2$ enrichment of cumulative GPP; cumulative NPP, cumulative nitrogen uptake; and cumulative nitrogen use efficiency calculated as cNPP/cNUP. The white bar is the median, the box the inter-quartile range, whiskers the range of data within four times the IQR, and dots are outliers.

# Supplementary Tables

Supplementary Table 1: BiomassCO2 model selection

| response | fixed effects | random group | random effects | AICc | method |
|---|---|---|---|---|---|
| $\Delta C_{veg}$ | (Intercept) | site | (Intercept) | 108.8 | ML |
| $\Delta C_{veg}$ | (Intercept), co2ELE | site | (Intercept) | 98.0 | ML |
| $\Delta C_{veg}$ | (Intercept), co2ELE | site | (Intercept), co2ELE | 100.0 | ML |
| $\Delta C_{veg}$ | (Intercept), co2ELE | site | (Intercept) | 97.0 | REML |
| $\Delta C_{veg}$ | (Intercept), co2ELE | site | (Intercept), co2ELE | 98.7 | REML |

Supplementary Table 2: meanNPP model selection

| response | fixed effects | random group | random effects | AICc | method |
|---|---|---|---|---|---|
| NPP | (Intercept) | site | (Intercept) | -24.8 | ML |
| NPP | (Intercept), eCO2 | site | (Intercept) | -42.2 | ML |
| NPP | (Intercept), eCO2 | site | (Intercept), eCO2 | -39.3 | ML |
| NPP | (Intercept), eCO2 | site | (Intercept) | -34.7 | REML |
| NPP | (Intercept), eCO2 | site | (Intercept), eCO2 | -31.7 | REML |

Supplementary Table 3: Biomass model selection

| response | fixed effects | random group | random effects | AICc | method |
|---|---|---|---|---|---|
| $\Delta C_{veg}$ | (Intercept) | site | (Intercept) | 108.8 | ML |
| $\Delta C_{veg}$ | (Intercept), cNPP, eCO2, cNPP:eCO2 | site | (Intercept) | 67.5 | ML |
| $\Delta C_{veg}$ | (Intercept), cNPP, eCO2 | site | (Intercept) | 65.5 | ML |
| $\Delta C_{veg}$ | (Intercept), eCO2 | site | (Intercept) | 98.0 | ML |
| $\Delta C_{veg}$ | (Intercept), cNPP, age[1], cNPP:age | site | (Intercept) | 63.5 | ML |
| $\Delta C_{veg}$ | (Intercept), cNPP, age, cNPP:age | site | (Intercept), cNPP | 66.1 | ML |
| $\Delta C_{veg}$ | (Intercept), cNPP, age | site | (Intercept) | 64.8 | ML |
| $\Delta C_{veg}$ | (Intercept), cNPP, age | site | (Intercept), cNPP | 62.6 | ML |
| $\Delta C_{veg}$ | (Intercept), cNPP | site | (Intercept) | 63.2 | ML |
| $\Delta C_{veg}$ | (Intercept), cNPP | site | (Intercept), cNPP | 60.2 | ML |
| $\Delta C_{veg}$ | (Intercept), cNPP | site | (Intercept) | 65.9 | REML |
| $\Delta C_{veg}$ | (Intercept), cNPP | site | (Intercept), cNPP | 61.1 | REML |

[1] Age since disturbance.

Supplementary Table 4: WoodAllocation model selection

| response | fixed effects | random group | random effects | AICc | method |
|---|---|---|---|---|---|
| fW | (Intercept) | site | (Intercept) | -73.6 | ML |
| fW | (Intercept), eCO2, cNPP, eCO2:cNPP | site | (Intercept) | -77.0 | ML |
| fW | (Intercept), eCO2, cNPP | site | (Intercept) | -80.2 | ML |
| fW | (Intercept), eCO2 | site | (Intercept) | -79.4 | ML |
| fW | (Intercept), cNPP | site | (Intercept) | -82.3 | ML |
| fW | (Intercept), cNPP | site | (Intercept), cNPP | -78.4 | ML |
| fW | (Intercept), cNPP | site | (Intercept) | -70.9 | REML |
| fW | (Intercept), cNPP | site | (Intercept), cNPP | -66.9 | REML |

Supplementary Table 5: ORNLWoodAllocation model selection

| response | fixed effects | parameter | se | t | P(t) | | F | dfn | dfd | P(F) | R2 | AICc |
|---|---|---|---|---|---|---|---|---|---|---|---|---|
| fW | (Intercept) | 0.6 | 0.0 | 15.3 | 0.0 | *** | 0.843 | 1 | 3 | 0.426 | 0.22 (-0.04) | 15.2 |
| | co2ELE | -0.1 | 0.1 | -0.9 | 0.4 | | | | | | | |

Supplementary Table 6: ORNLRootAllocation model selection

| response | fixed effects | parameter | se | t | P(t) | | F | dfn | dfd | P(F) | R2 | AICc |
|---|---|---|---|---|---|---|---|---|---|---|---|---|
| fFR | (Intercept) | 0.1 | 0.0 | 2.6 | 0.1 | . | 1.59 | 1 | 3 | 0.296 | 0.35 (0.13) | 15.4 |
| | co2ELE | 0.1 | 0.1 | 1.3 | 0.3 | | | | | | | |
